# Re-appraisal of Standard of Care Imaging to Identify Predictors of Treatment Outcomes among Patients with Bladder and Upper Tract Urothelial Cancers

**DOI:** 10.1007/s11934-025-01278-0

**Published:** 2025-06-21

**Authors:** Shelby Harper, Erick M. Remer, Nima Almassi

**Affiliations:** 1 Department of Urology, Glickman Urological and Kidney Institute, Cleveland Clinic, Cleveland, USA; 2 Diagnostics Institute, Cleveland Clinic, Cleveland, USA

**Keywords:** Bladder cancer, Cystectomy, Sarcopenia, Neoadjuvant therapy, Treatment outcomes, Biomarkers

## Abstract

**Purpose of Review:**

Urothelial carcinoma is a prevalent malignancy within the United States that may involve the upper and/or lower urinary tracts. Multimodal treatment is often employed, with transurethral resection and intravesical therapy standard of care for non-muscle-invasive disease; neoadjuvant systemic therapy followed by radical cystectomy or trimodal therapy for muscle-invasive disease; and combination immune checkpoint inhibitors and antibody-drug conjugates standard of care for metastatic disease. These treatments carry risks of surgical complication or treatment-associated toxicity which can impair quality of life. Predictive biomarkers of treatment tolerability are currently limited.

**Recent Findings:**

There is emerging evidence that radiological biomarkers can predict treatment outcomes among patients with urothelial carcinoma.

**Summary:**

In this review, we evaluate the existing data on radiological biomarkers evaluable from current standard-of-care imaging in predicting treatment outcome among patients with urothelial carcinoma.

## Introduction

Urothelial carcinoma is the 6th most common malignancy in the United States as of 2024 [1], though it is impossible to quantify the financial, personal, and emotional toll on patients. Urothelial carcinoma most commonly originates in the bladder, with up to 5–10% developing in the upper urinary tract [1]. Commonly presenting with either microscopic or gross hematuria, the standard of care evaluation for urothelial malignancy includes upper tract imaging and endoscopic assessment with cystoscopy and/or ureteroscopy per AUA guidelines. Radiographically, contrast-enhanced imaging, preferably computed tomographic urography (CTU), is recommended during the initial diagnostic work-up and as part of surveillance following treatment [2]. The National Comprehensive Cancer Network (NCCN) stratifies urothelial carcinoma of the bladder into three categories: non-muscle invasive bladder cancer (NMIBC), muscle-invasive bladder cancer (MBIC), and metastatic urothelial carcinoma [2]. Upper tract urothelial carcinoma (UTUC) remains a difficult entity to fully risk stratify due to limitations with tissue sampling and diagnostic imaging in accurately staging disease [3]. Treatment paradigms for urothelial carcinoma are dependent on a number of factors, including tumor grade, clinical stage, disease focality, and a patient’s overall health status. Across disease stages, multimodal treatment is typically employed: NMIBC is typically treated with endoscopic surgical resection and intravesical therapy; MIBC is treated with neoadjuvant chemotherapy (NAC) followed by radical cystectomy (RC) or trimodal therapy (TMT) consisting of endoscopic resection followed by chemoradiation; while systemic therapy with immune checkpoint inhibitors, antibody-drug conjugates, and chemotherapy are standard of care treatments for metastatic disease. Unfortunately, toxicity from systemic therapy or radiation and complications and quality of life impacts from major urologic surgery are known sequelae of treatment. Treatment toxicity can impact a patient’s ability to complete treatment, potentially compromising oncologic outcomes, and can negatively impact quality of life. Identifying predictors of treatment tolerability and response remains an area of need as biomarkers are currently limited. In this review, we evaluate the existing data on radiological biomarkers evaluable from current standard-of-care imaging in predicting treatment outcome among patients with urothelial carcinoma.

### Sarcopenia– Definition, Diagnostic Criteria, and Clinical Relevance

Sarcopenia is defined as skeletal muscle deficiency, marked by the progressive decline in skeletal muscle mass and strength. This has traditionally been viewed as an age-related phenomenon. Pathophysiologically, sarcopenia is fundamentally an imbalance of anabolic and catabolic processes, resulting in fatty infiltration and ultimately decline in skeletal muscle volume and strength [4]. While advancing age has been definitively associated with the development of sarcopenia, debilitation, immobility, and chronic disease states also promote this process. For example, there is interplay between sarcopenia and cardiovascular disease, with sarcopenia worsening cardiovascular outcomes and cardiovascular disease promoting the development of sarcopenia [5]. Further, sarcopenia has been associated with an increased risk of falls and fractures [6], cognitive impairment [7], as well as lower quality of life and loss of independence [8, 9]. In large cross-sectional population-based studies, sarcopenia has been shown to be an independent risk factor for all-cause mortality, highlighting its clinical importance as a prognostic biomarker [10]. 

Given the significant impact of sarcopenia on the aging population, there has been growing interest in evaluating the impact of sarcopenia on treatment outcomes among cancer patients. Strength assessment is a necessary component of evaluating muscle function and can be non-invasively performed with a series of physical tests, questionnaire scales, and anthropometrics [11, 12]. As strength assessments are not routinely performed in clinical practice across oncologic subspecialties, much of the literature uses skeletal muscle mass as a surrogate for sarcopenia. Skeletal muscle mass can be quantified with imaging modalities including dual-energy X-ray absorptiometry (DEXA), CT, MR, and ultrasound (US) [13]. With DEXA and US demonstrating more variability due to dependence on hydration status and poor reproducibility, respectively, CT and MR have been more widely utilized in the published literature. Many studies quantify skeletal muscle mass by calculating the skeletal muscle index (SMI), defined as the cross-sectional area of skeletal muscle at the L3 vertebral level (cm^2^) divided by patient height in meters squared (m^2^) [14]. SMI can be measured reproducibly using standard-of-care cross-sectional staging imaging for intraabdominal or pelvic malignancies without requiring significant added work. However, a recurring criticism of using SMI or other radiographic-based measured as a surrogate for sarcopenia is that it does not account for muscle function or strength. Despite these limitations, studies across surgical disciplines have demonstrated a strong association between skeletal muscle deficiency and adverse post-operative outcomes [15–17]. To minimize interobserver variability and automate body composition analysis in abdominal imaging, machine learning and artificial intelligence (AI) algorithms have been developed to automate image segmentation and body composition analysis with high accuracy [18–20]. These algorithms can facilitate accurate muscle mass quantification and assess myosteatosis which may be another clinically-relevant marker of sarcopenia. Implementing such models in routine diagnostic imaging practice could generate larger datasets for prospective study while also allowing real-time identification of patients with skeletal muscle deficiency [21], which can predict treatment outcomes and potentially help guide management as we examine later in this review.

### Sarcopenia and Outcomes in Patients with Bladder Cancer Undergoing Radical Cystectomy

Radical cystectomy (RC) remains a standard of care treatment option for MIBC, BCG-unresponsive NMIBC, and very high-risk NMIBC with variant histology. RC carries significant risk of perioperative complications, with risk of 90-day post-operative mortality approaching 5% in some studies [18–25]. Regardless of surgical approach, RC is associated with significant risk of reduced physical activity and worsened disability in the early post-operative period, although the ultimate long-term quality of life outcomes are overall favorable [26, 27]. Biomarkers which could predict postoperative recovery and surgical outcomes would be clinically useful to guide patient counseling, assess surgical candidacy, and to inform treatment selection.

In an effort to determine the impact of sarcopenia on survival outcomes after RC, Psutka and colleagues evaluated cancer-specific and all-cause mortality among 205 patients after RC [17]. Sarcopenia was defined using sex-specific consensus definitions of SMI measured on preoperative abdominal CT imaging. Of this cohort, 69% of patients were classified as sarcopenic based on international consensus definitions. Sarcopenic patients demonstrated worse 5-year cancer-specific and overall survival compared to those without sarcopenia. These observations have been corroborated in subsequent studies and are well-summarized in a recent meta-analysis [28]. 

In addition to its prognostic significance, sarcopenia also appears predictive of early, adverse post-operative outcomes. Observational studies have demonstrated sarcopenia to be associated with a higher risk of postoperative complications, major complications, and discharge to a nursing facility following surgery [29–32]. This is likely related to the impact of skeletal muscle deficiency on a patient’s overall fitness and ability to withstand physiological stress. In the context of major surgery such as RC, sarcopenia may reflect a patient’s physiologic reserve to withstand the stress of major surgery. The strong association between sarcopenia and adverse post-operative outcomes raises the question as to whether preoperative interventions to reverse sarcopenia would improve treatment outcomes in cancer patients. Given the importance of time-to-treatment in optimizing oncologic outcomes [33], such interventions would have to be quickly implemented and demonstrate rapid effect.

Preoperative fitness assessments may represent one method of identifying and potentially “prehabilitating” high-risk, sarcopenic patients. Unfortunately, preoperative fitness assessment and prehabilitation programs are not standard across surgical practices. Some groups use simple metrics such as baseline comorbidities [34] or serum albumin levels [35] to screen for frailty or malnutrition. Others have implemented more comprehensive fitness assessments in the preoperative evaluation of patients undergoing RC. For example, investigators from Memorial Sloan Kettering Cancer Center developed and implemented utilization of an electronic Rapid Fitness Assessment (eRFA) tool in a cohort of geriatric patients over age 75 undergoing RC. The eRFA is a web-based geriatric assessment tool which evaluates 13 domains, including measures of functional ability and social support, to determine overall frailty. Patients were assessed prior to RC and followed for adverse outcomes. Patients with higher eRFA scores were more likely to require intensive care unit admission, die within 90 days, or be discharged to a facility.[36] Such tools can help identify at-risk patients who may be most likely to benefit from pre-surgical intervention.

Identifying effective “prehabilitation” interventions for patients identified as high-risk based on the presence of sarcopenia, eRFA assessment, nutritional screening, or other biometrics, remains an area of need. In a pilot study assessing the feasibility and impact of a “prehabilitation” program for patients undergoing RC, investigators implemented smoking cessation counseling, malnutrition screening and nutritional supplementation, prescribed exercise and physical therapy, and onco-psychology screening [37]. Implementation of this program was feasible, although with widely varied rates of compliance in completion of the different domains of the prehabilitation program. However, the impact of this program on post-operative outcomes at this time remains undefined. In a separate, randomized trial of preoperative nutritional intervention before RC, nutritional supplementation resulted in less post-operative weight loss and a lower incidence of postoperative sarcopenia, however differences in the incidence of postoperative complications and readmission were not statistically-significant between groups [38]. 

Taken together, the existing literature suggests radiological, serum-based, and anthropometric measures can help identify patients with deficiencies of strength, skeletal muscle, and nutrition, all of which have been shown to be predictive of adverse outcomes after RC. Further work is needed to determine effective interventions to mitigate these risks.

### Impact of Sarcopenia on Tolerability and Treatment Outcomes with Neoadjuvant Chemotherapy

Among patients with MIBC, neoadjuvant chemotherapy (NAC) prior to RC has been shown to confer a survival benefit and is considered the standard of care for cisplatin-eligible patients. The landmark SWOG 8710 randomized control trial of NAC followed by RC versus RC alone for treatment of MIBC demonstrated superior overall survival among patients who received three cycles of neoadjuvant Methotrexate, Vinblastine, Adriamycin, and Cisplatin (MVAC) followed by RC [39]. This pivotal study highlighted the benefit of the alkylating agent, cisplatin, in the treatment of urothelial carcinoma. Unfortunately, the classic MVAC regimen carries significant toxicity, with up to 63% of patients requiring a dose reduction or cessation of therapy due to adverse effects [40]. Identification of this limitation spurred interest in alternative regimens. Work from the Netherlands found similar pathologic response rates of dose-dense MVAC (ddMVAC), delivered at shorter treatment intervals, to that of traditional MVAC therapy, with reduced toxicity [41]. Several observational studies suggested similar efficacy with neoadjuvant Gemcitabine and Cisplatin (GC), although the VESPER clinical trial demonstrated superior progression-free survival among patients treated with six cycles of ddMVAC compared to four cycles of GC [42]. 

ddMVAC and GC remain the most commonly utilized neoadjuvant chemotherapy regimens for MIBC, however they are not options for patients with renal insufficiency given cisplatin’s dependency on renal clearance. In clinical practice, the renal function cutoff to determine cisplatin eligibility varies. The Galsky criteria include a creatinine clearance of > 60mL/min as one criterion for cisplatin eligibility [43], however in clinical practice patients with glomerular filtration rate (GFR) of 50–60 ml/min are routinely treated with cisplatin-based NAC with adjunctive measures including aggressive hydration. Treatment of such patients does carry a higher risk of premature treatment discontinuation or need for dose modification due to renal toxicity [44]. 

The survival benefit of NAC must be weighed against the risk of treatment toxicity which may render a patient unable to proceed with RC, an outcome impacting up to 15% patients in some trials [39]. The stress-mediated catabolic response to NAC may also induce or potentiate pre-existing sarcopenia which may further negatively impact treatment outcomes [45]. Zagar and colleagues explored this idea by examining skeletal muscle loss after treatment with NAC and its impact on treatment tolerability and pathologic outcomes. Skeletal muscle loss was identified by measuring changes in psoas muscle volume on pre- and post-NAC CT imaging. These investigators observed psoas muscle volume loss following cisplatin-based NAC to be predictive of NAC dose reduction or treatment delay; however, psoas muscle loss was not associated with lower pathologic response rates or higher risk of surgical complication or postoperative readmission [46]. Similar work by Lyon and colleagues demonstrated significant decline in SMI with NAC, although neither baseline sarcopenia or degree of skeletal muscle loss with NAC was associated with pathologic down-staging (≤ ypT1N0) or complete pathologic response (ypT0N0) [47]. These results suggest that although treatment with NAC impacts body composition and promotes skeletal muscle loss, sarcopenia is not predictive of pathologic response or oncologic outcomes following treatment with NAC and RC.

More recent work has sought to determine whether sarcopenia may predict tolerability or risk of toxicity of treatment with NAC. Although creatinine clearance > 60 mL/min was originally felt to be a criterion for cisplatin eligibility, patients with GFR 50–60 mL/min can be administered cisplatin with split dosing and aggressive hydration at the potential cost of a greater risk of toxicity [43]. Accurately determining GFR in such patients with “borderline” renal function for cisplatin is therefore crucial. While plasma clearance of iohexol is considered a gold standard method of measuring renal function, it is costly and difficult to implement in clinical practice. Therefore, GFR is commonly estimated using creatinine-based equations, such as the Chronic Kidney Disease Epidemiology Collaboration (CKD-EPI) equation. However, these equations do not account for body composition or muscle turnover. There is evidence that renal function is overestimated by such equations in patients with skeletal muscle deficiency or diminished muscle turnover, which may have important implications for bladder cancer patients in whom sarcopenia is highly prevalent. In a study of non-weight bearing patients with neurogenic lower urinary tract dysfunction, Werneburg and colleagues demonstrated significantly higher estimated GFR using creatinine-based equations compared to equations utilizing cystatin C, an endogenous filtration marker that is not dependent on skeletal muscle mass.[48] These observations suggest that creatinine-based methods of estimating renal function, which are widely used in practice, may not be accurate in patients with skeletal muscle deficiency.

Based on these observations, we hypothesized that baseline renal function may be overestimated with traditional eGFR calculations in patients with bladder cancer, given the high prevalence of sarcopenia in these patients. For patients with “borderline” renal function for cisplatin, overestimating renal function may leave such patients at higher risk of NAC-associated toxicity. To test this hypothesis, we evaluated a cohort of 216 patients with MIBC treated with NAC and RC. SMI was determined using baseline staging scans, with 83% of patients meeting consensus definition for sarcopenia based on sex-specific SMI cutoffs. Half of the patients in this cohort had “borderline” renal function for cisplatin, which in this study was defined as an eGFR of 40–65 mL/min using creatinine-based equations. Sarcopenia was found to be an independent predictor of NAC-associated renal toxicity. Furthermore, sarcopenia appeared to confer a greater risk of NAC-associated renal toxicity among patients with “borderline” renal function for cisplatin compared to patients with an eGFR ≥ 65 mL/min.[49] These observations suggest that sarcopenia increases risk of cisplatin-associated toxicity among patients with borderline renal function. Further work is needed to determine optimal methods of estimating renal function especially among patients with sarcopenia to best determine cisplatin eligibility. Clinical trials are currently in development to compare creatinine-based and cystatin C-based eGFR calculations against measured GFR as a gold standard. As immunotherapy and novel systemic therapy agents including antibody-drug conjugates become increasingly utilized, additional research is needed to determine whether sarcopenia or body composition is associated with treatment efficacy or tolerability with these agents.

### Body Composition and Wound-Related Outcomes after Abdominal Surgery

Body composition analysis using standard of care staging or surveillance imaging can identify other morphometric features that may be predictive of surgical and quality of life outcomes. For example, among patients undergoing RC with ileal conduit urinary diversion, parastomal hernia (PH) is a common complication which can negatively impact body image and overall quality of life. Until recently, the existing literature focused on medical comorbidities including obesity, malnutrition, and active smoking as risk factors for PH. To determine whether other aspects of body composition beyond body mass index (BMI) impact PH risk, Lone and colleagues conducted an observational study of 367 patients who underwent RC with ileal conduit urinary diversion at Cleveland Clinic. Body composition analysis was performed, measuring SMI and fat mass index (FMI) using preoperative staging scans. Increasing FMI and decreasing SMI were predictive of PH in logistic regression analysis in this study [50]. If validated, these findings suggest body composition analysis using standard of care preoperative imaging could help guide patient counseling and potentially guide interventional trials to mitigate PH risk in these patients.

The current literature demonstrates limited efficacy to pre- or peri-operative interventions to reduce risk of PH following RC. There has been interest in the use of prophylactic parastomal mesh placement based on efficacy in the colorectal surgery literature; [51, 52] however, evidence supporting efficacy of prophylactic parastomal mesh placement at the time of RC with ileal conduit urinary diversion is conflicting. A Swedish trial of 242 patients undergoing open RC with ileal conduit diversion demonstrated a significantly lower risk of PH hernia among patients receiving prophylactic mesh (23% PH incidence in the control arm vs. 11% in the prophylactic mesh arm, HR 0.45, 95% CI 0.24–0.86) [53]. Conversely, two trials conducted at the University of Southern California (146 patients) and Memorial Sloan Kettering Cancer Center (178 patients), respectively, demonstrated no benefit to prophylactic mesh placement [53, 54]. It is possible that prophylactic mesh, or other interventions, may confer benefit to high-risk patients as determined by body composition analysis, although more study is needed to identify effective means of reducing PH risk in these patients.

### Opportunistic Screening

Opportunistic screening through standard of care imaging can facilitate diagnosis of clinically important conditions that may impact patient management and outcomes. For example, osteoporosis and osteopenia are increasingly prevalent in the aging population. Although patients with bladder and upper urinary tract urothelial carcinoma often have multiple risk factors for bone density loss such as increased age, post-menopausal status, and smoking history, it remains underdiagnosed in this population and is not a component of standard work-up for these patients. Furthermore, there is evidence that some components of cancer treatment may increase risk of fragility fracture. For example, RC with urinary diversion increases the risk of metabolic acidosis which can promote bone resorption, bone density loss, and increase fracture risk. In a SEER-Medicare study of over 50,000 bladder cancer patients, treatment with RC was associated with a 21% greater risk of any fracture [55]. Corticosteroids, which are commonly used to treat immune-mediated toxicity of immune checkpoint inhibitors, also increases fracture risk [53–55]. With level I evidence supporting the efficacy of bone targeted agents in reducing risk of fracture in patients with osteoporosis and osteopenia [59, 60], identifying patients at elevated fracture risk at baseline would allow for early treatment with prophylactic therapy. Given the significant morbidity and increased risk of mortality associated with osteoporotic fracture [61], early diagnosis through opportunistic screening to allow early prophylactic treatment represents one strategy to improve clinical and quality of life outcomes among patients with urothelial carcinoma.

DEXA is the preferred imaging modality for assessing bone mineral density (BMD), diagnosing osteoporosis and osteopenia, and estimating fracture risk. Additionally, DEXA has the capability of obtaining full-body composition, including assessment of lean body mass and fat mass [62]. Despite these advantages, DEXA can be time-consuming, require expertise, and incur added cost, which may hinder routine use in a clinical oncology practice. When considering the necessity of pre-operative imaging prior to RC or radical nephroureterectomy, opportunistic screening for osteoporosis or osteopenia using standard of care imaging is appealing. Deshpande and colleagues conducted a systematic review of the literature to evaluate the efficacy of CT imaging compared to DEXA as a reference standard in identifying osteoporosis. Vertebral body attenuation < 100 Hounsfield units (HU) was found to be highly predictive of osteoporosis while vertebral body attenuation > 160 HU was associated with a significantly lower risk of osteoporosis [63]. Furthermore, there is some evidence that quantitative CT may actually have higher sensitivity in detecting osteoporosis than DEXA, which may be confounded by factors such as abdominal vascular calcifications [64]. In a study of over 9000 asymptomatic adult patients undergoing abdominal CT imaging, Pickhardt and colleagues demonstrated automated bone and muscle attenuation to have similar performance characteristics to the Fracture Risk Assessment Tool (FRAX) in predicting future osteoporotic fracture [65]. Although there are no studies to date evaluating the utility of preoperative imaging for opportunistic osteoporosis screening among patients with urothelial carcinoma, the existing literature suggests this may facilitate identification of at-risk patients who may benefit from prophylactic treatment. Given the increased risk of fracture among patients undergoing RC, studies are warranted to evaluate the feasibility and efficacy of such screening in reducing fracture risk in these patients.

## Conclusion

Urothelial carcinoma is a prevalent malignancy within the United States, requiring long-term interdisciplinary management. Sarcopenia is highly prevalent in this patient population and portends worse outcomes following treatment with surgery and systemic therapy. There is emerging evidence that body composition analysis using preoperative staging imaging can identify patients at risk of post-operative complication, readmission, and treatment-related long-term sequelae including PH. Opportunistic screening using staging imaging can also allow earlier diagnosis of osteoporosis or osteopenia, which are highly prevalent yet underdiagnosed among patients with urothelial carcinoma, allowing earlier prophylactic treatment to reduce risk of fracture-related morbidity. Implementing body composition analysis in routine practice may help identify “at-risk” patients who may benefit from pre-treatment prehabilitation, which remains a focus on clinical investigation. Furthermore, sarcopenia may predict higher risk of toxicity with cisplatin-based chemotherapy among patients with “borderline” renal function for cisplatin, likely due to over-estimation of renal function using creatinine-based calculations in these patients. Ongoing studies are evaluating accuracy of alternate methods of renal functional assessment to better identify candidates for cisplatin. As alternate systemic therapies including immune checkpoint inhibitors and antibody drug conjugates become increasingly utilized, further research is needed to determine whether sarcopenia or other radiologic biomarkers are associated with treatment efficacy or tolerability.

## Key References

- Chesnut GT, Tin AL, Sjoberg DD et al. Electronic Rapid Fitness Assessment Identifies Factors Associated with Adverse Early Postoperative Outcomes following Radical Cystectomy. J Urol. 2021;205(2):400–406. 10.1097/JU.0000000000001360
-
  - Prospective study demonstrating use of an electronic rapid fitness assessment is predictive of adverse post-operative outcomes among geriatric patients undergoing cystectomy.
- Werneburg GT, Hettel D, Jeong S, Nemunaitis G, Taliercio JJ, Wood HM. Estimated Glomerular Filtration Rate Using Cystatin C is a More Sensitive Marker for Kidney Dysfunction in Nonweight-bearing Individuals. J Urol. 2023;209(2):391–398. 10.1097/JU.0000000000003070
-
  - Observational study demonstrating the impact of skeletal muscle deficiency on estimated glomerular filtration rate using creatinine-based calculations.
  - This study found that serum creatinine and cystatin C were not equivalent in accurately estimating renal function in nonweight-bearing individuals.
- Haile, Lone ES, Shin Z et al. D, Sarcopenia may increase cisplatin toxicity in bladder cancer patients with borderline renal function. BJU Int. 2024. 10.1111/BJU.16606
-
  - Observational study demonstrating sarcopenia as a predictor of neoadjuvant chemotherapy-associated toxicity, and suggesting that sarcopenia further increases risk of renal-associated toxicity of neoadjuvant chemotherapy among patients with "borderline" renal function for cisplatin.

## Data Availability

No datasets were generated or analysed during the current study.
